# ANGPTL7 is transcriptionally regulated by SP1 and modulates glucocorticoid-induced cross-linked actin networks in trabecular meshwork cells via the RhoA/ROCK pathway

**DOI:** 10.1038/s41420-022-00847-3

**Published:** 2022-02-08

**Authors:** Mengsha Sun, Wenjia Liu, Minwen Zhou

**Affiliations:** 1 Department of Ophthalmology, Shanghai East Hospital, Tongji University School of Medicine, Shanghai, China; 2 Department of Ophthalmology, Shanghai General Hospital (Shanghai First People’s Hospital), Shanghai Jiao Tong University School of Medicine, Shanghai, China; 3 National Clinical Research Center for Eye Diseases, Shanghai, China; 4 Shanghai Key Laboratory of Fundus Diseases, Shanghai, China

**Keywords:** Glaucoma, Actin

## Abstract

Glaucoma is one of the leading causes of worldwide irreversible blindness. Lowering elevated intraocular pressure (IOP) is currently the only effective approach for controlling the progress of glaucoma. Angiopoietin-like 7 (ANGPTL7) takes a key part in elevated outflow resistance of aqueous humor in dysfunctional trabecular meshwork (TM), along with the formation of cross-linked actin networks (CLANs), leading to high IOP. In this study, we explored the role of the ANGPTL7 signaling pathway in CLAN formation. We detected the expression of ANGPTL7 in cultured primary TM cells treated with dexamethasone (DEX) and ethanol as a control using qRT-PCR and western blotting. Actin filaments were revealed by phalloidin staining. ANGPTL7 short hairpin RNA (shRNA) was applied to TM cells to examine the effect of ANGPTL7 on DEX-induced CLAN formation. Western blotting was used to assess the effect of ANGPTL7 on the RhoA/Rho-associated kinase (Rho-kinase/ROCK) signaling pathway. Bioinformatics, dual-luciferase reporter assays, and chromatin immunoprecipitation were employed to identify the transcription factors of ANGPTL7. Transcription factor specificity protein 1 (SP1) overexpression and silencing were performed to determine their roles in the modulation of ANGPTL7 expression. We found DEX-induced ANGPTL7 expression and stress fiber rearrangement in TM cells. ANGPTL7 knockdown effectively inhibited the formation of CLANs. Moreover, it was involved in the regulation of the RhoA/ROCK signaling pathway, further affecting DEX-induced CLAN formation. SP1 was identified as a transcription factor of ANGPTL7 which regulated ANGPTL7 level to mediate CLAN formation through the RhoA/ROCK signaling pathway. This study contributes to revealing the molecular mechanisms of ANGPTL7 in CLAN formation, which is involved in TM dysfunction and glaucoma pathogenesis.

## Introduction

Glaucoma is a leading cause of worldwide irreversible vision loss, characterized by progressive optic neuropathy [1]. The most common form of glaucoma is primary open-angle glaucoma (POAG), which is always accompanied by high intraocular pressure (IOP), the key risk factor for the pathogenesis of POAG [2]. Interestingly, prolonged use of dexamethasone (DEX) poses a high risk of elevated IOP and results in secondary glaucoma, which has many common characteristics with POAG [3, 4]. Hence, the pathogenesis of POAG can be deduced from the mechanisms underlying DEX-induced ocular hypertension. Understanding the DEX-induced molecular mechanisms may assist in developing therapies for glucocorticoid-induced glaucoma and POAG.

High IOP is caused by increased outflow resistance of aqueous humor (AH) [5]. Accumulating evidence suggests that actin cytoskeletal rearrangement of the trabecular meshwork (TM) forming cross-linked actin networks (CLANs) is a crucial contributor to this increased resistance [6–8]. CLANs are found mainly in TM cells both in vivo and in vitro which form a geodesic network with polygonal actin arrangements. There is inadequate knowledge of the molecular mechanisms underlying CLAN formation. Therefore, the molecular mechanisms of CLAN formation warrants investigation.

Previous studies have found that the concentration of angiopoietin-like 7 (ANGPTL7) is increased in glaucomatous AH and that overexpression of ANGPTL7 in the TM alters the components of the extracellular matrix (ECM) [9, 10]. A recent study found that ANGPTL7 protein-altering variants exert a strong protective effect on glaucoma and suggested ANGPTL7 as a therapeutic target for glaucoma [11]. Thus, ANGPTL7 is likely to play a vital role in modulating TM’s ECM and regulating IOP.

It is imperative to elucidate the molecular mechanisms of ANGPTL7 that lead to glaucoma pathology and find new potential therapeutic targets. A previous study reported that transcription factor specificity protein 1 (SP1) is involved in promoter activity and further regulates the F-actin architecture of the Schlemm’s canal endothelium, suggesting that SP1 may function in the development and progression of glaucoma [12]. In this study, we identified the potential transcription factors of ANGPTL7 using bioinformatics and selected SP1 as an upstream molecule for further study. Moreover, we chose the RhoA/Rho-associated kinase (Rho-kinase/ROCK) signaling pathway based on bioinformatics analyses and previous reports on its functions in cytoskeletal reorganization and stress fiber formation [13, 14]. Then, we determined the molecular mechanisms of ANGPTL7 in CLAN formation by conducting a series of biological experiments.

## Results

### Dexamethasone induced ANGPTL7 expression in TM cells

**Fig. 1:**
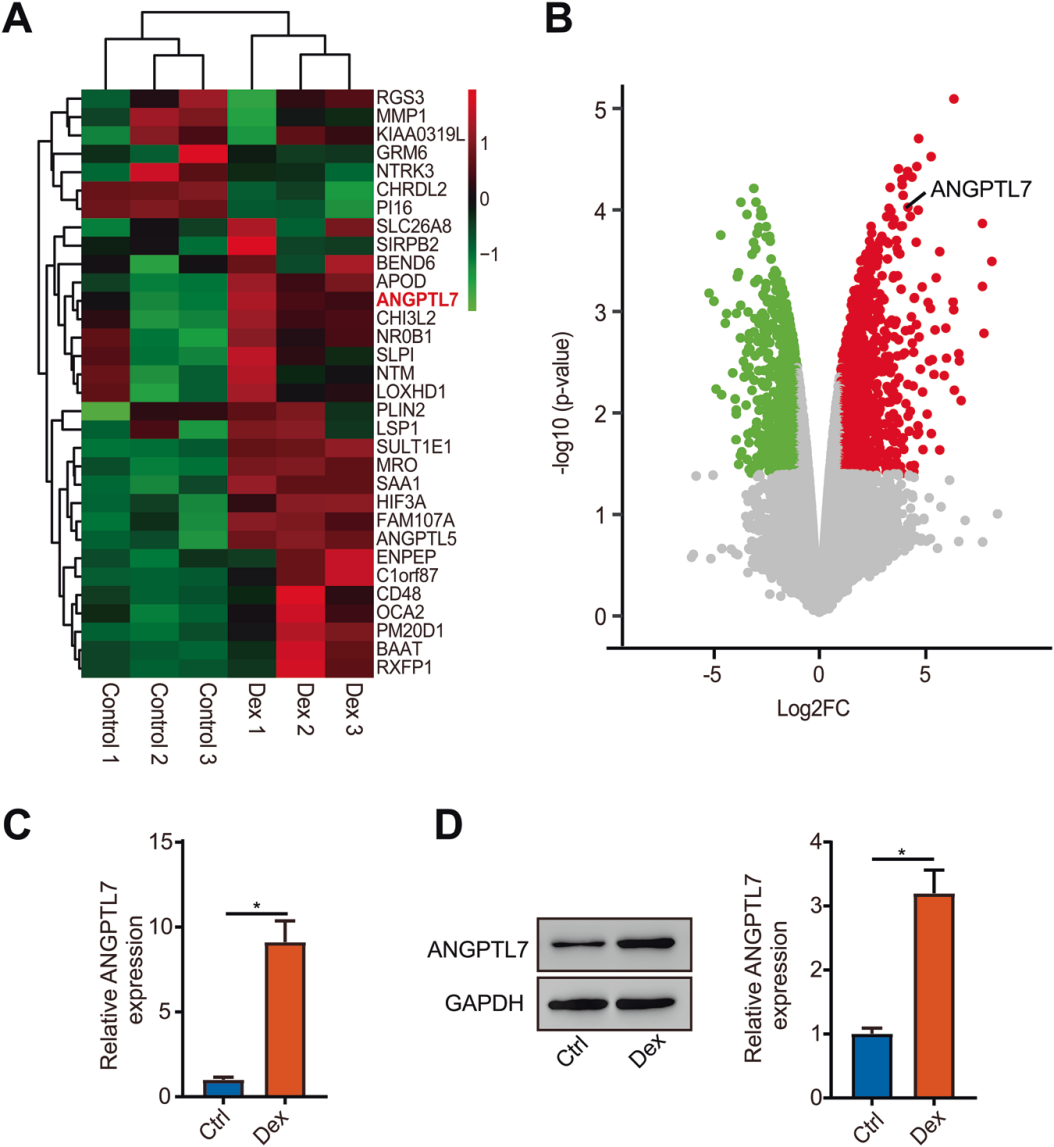
Dexamethasone (DEX) induced ANGPTL7 expression in trabecular meshwork (TM) cells. **A** Heatmap of differentially expressed genes by analysis of TM cells treated with and without 100 nM DEX for 14 days from GSE65240. ANGPTL7 expression was distinctly upregulated with DEX treatment in TM cells. **B** Volcano plot of differentially expressed genes. ANGPTL7 is marked out. The red dots indicate distinctly upregulated genes. Fold change ≥ 2, *P* < 0.05. TM cells were treated with DEX or a solvent (ethanol) as a control for 14 days. **C** Messenger RNA and **D** protein expression of ANGPTL7 detected by qRT-PCR and western blotting showed high levels with DEX treatment. *N* = 3 from three biological replicates. ^*^P < 0.05.

To assess the expression of steroid-induced glaucoma and POAG-related genes, the expression data from the GEO database (GSE65240) were analyzed first. The heatmaps and volcano plots identifying the differentially expressed genes showed that ANGPTL7 expression was distinctly upregulated in the TM cells with DEX treatment (Fig. 1A, B). Next, the messenger RNA (mRNA) expression of ANGPTL7 was tested by qRT-PCR, and the protein expression of ANGPTL7 was examined by WB. The results showed high levels of ANGPTL7 with DEX treatment (Fig. 1C, D).

### ANGPTL7 regulated DEX-induced CLAN formation

**Fig. 2:**
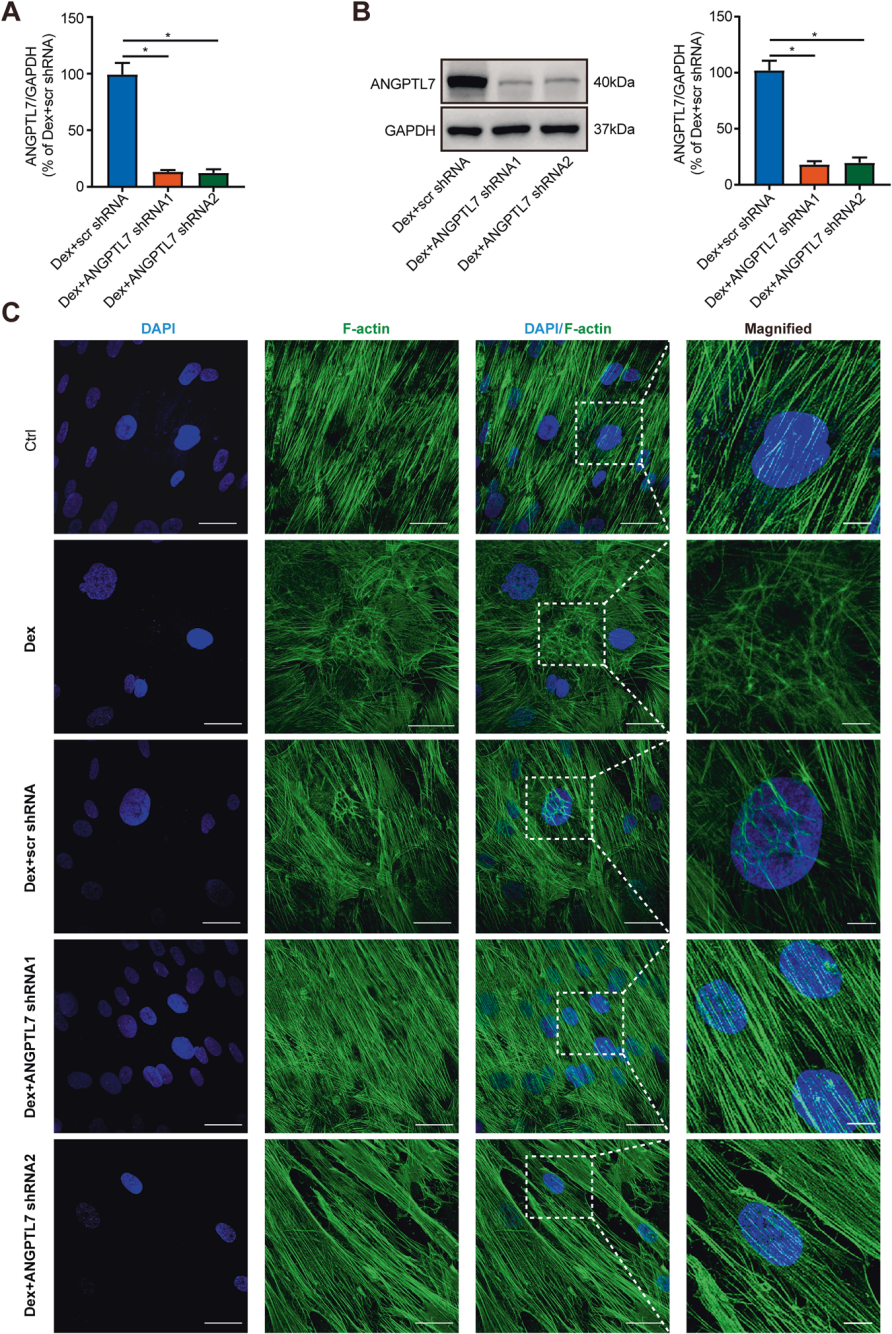
ANGPTL7 regulated DEX-induced CLAN formation. After TM cells were transfected with scr shRNA and ANGPTL7 shRNAs (shRNA1, shRNA2) and treated with DEX, the **A** mRNA expression and **B** protein expression of ANGPTL7 were measured by qRT-PCR and western blotting. ANGPTL7 shRNAs (shRNA1, shRNA2) substantially reduced the expression of ANGPTL7 compared with scr shRNA. **C** Representative images of actin filaments at the perinuclear region (60× magnification). The insets show regions at higher magnification (200×). Digital zoom revealed the detailed actin structure. TM cells formed dome-like structures with DEX treatment. The formation of CLANs in the DEX + scr shRNA group resembled that in the DEX-treated group. Conversely, in the DEX + ANGPTL7 shRNAs (shRNA1, shRNA2) groups, CLAN formation was significantly reduced. Blue: DAPI; green: F-actin. *N* = 3 from three biological replicates. ^*^P < 0.05.

To study the effect of ANGPTL7 on CLAN formation, we first identified the transfection effect of ANGPTL7 shRNA by measuring the mRNA and protein expressions of ANGPTL7 in TM cells which were transfected with scr shRNA and ANGPTL7 shRNAs (shRNA1, shRNA2) before DEX treatment. The results showed that both ANGPTL7 shRNA1 and ANGPTL7 shRNA2 substantially reduced the expression of ANGPTL7 compared with scr shRNA (Fig. 2A, B). IF staining showed that TM cells formed dome-like structures in the DEX-treated group. The formation of CLANs in the DEX + scr shRNA group resembled that in the DEX-treated group. Conversely, in the DEX + ANGPTL7 shRNAs (shRNA1, shRNA2) group, CLAN formation was significantly reduced(Fig. 2C). This indicated that DEX-induced CLAN formation, while ANGPTL7 knockdown suppressed it.

### ANGPTL7 modulated CLAN formation via the RhoA/ROCK pathway

**Fig. 3:**
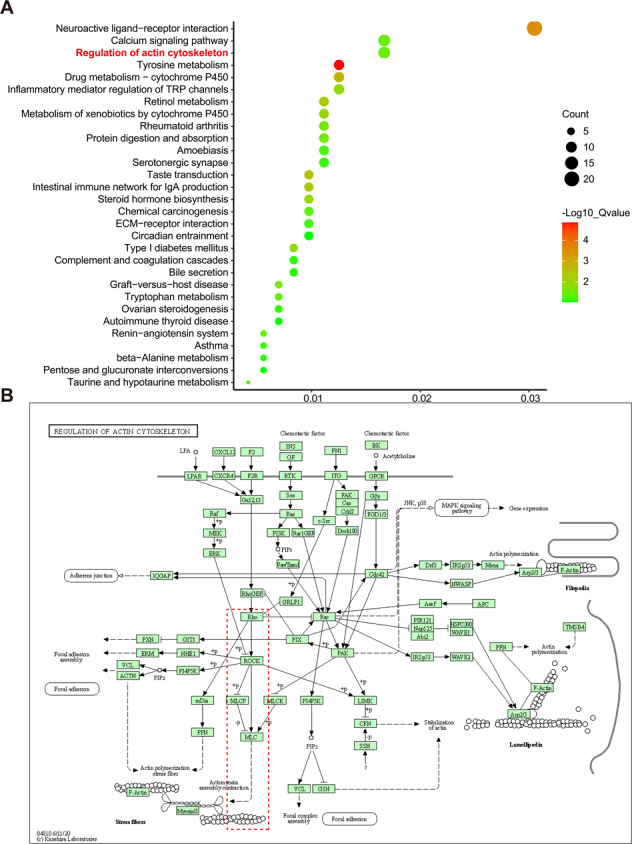
The RhoA/ROCK pathway in regulation of actin cytoskeleton. **A**, **B** Based on KEGG pathway enrichment analysis, the differentially expressed genes were enriched in the regulation of actin cytoskeleton pathway. In this pathway diagram, Rho, ROCK, and MLC were the key genes in the RhoA/ROCK pathway.

The KEGG pathway enrichment analysis of GSE65240 showed that differentially expressed genes took part in some pathways, such as calcium signaling pathway, neuroactive ligand-receptor interaction, tyrosine metabolism, and regulation of actin cytoskeleton (Fig. 3A). Actin cytoskeleton pathway which is associated with cytoskeleton reorganization might participate in CLAN formation. As the RhoA/ROCK pathway was an important part of the regulation of actin cytoskeleton (Fig. 3B), we selected it as a potential pathway downstream of ANGPTL7.

**Fig. 4:**
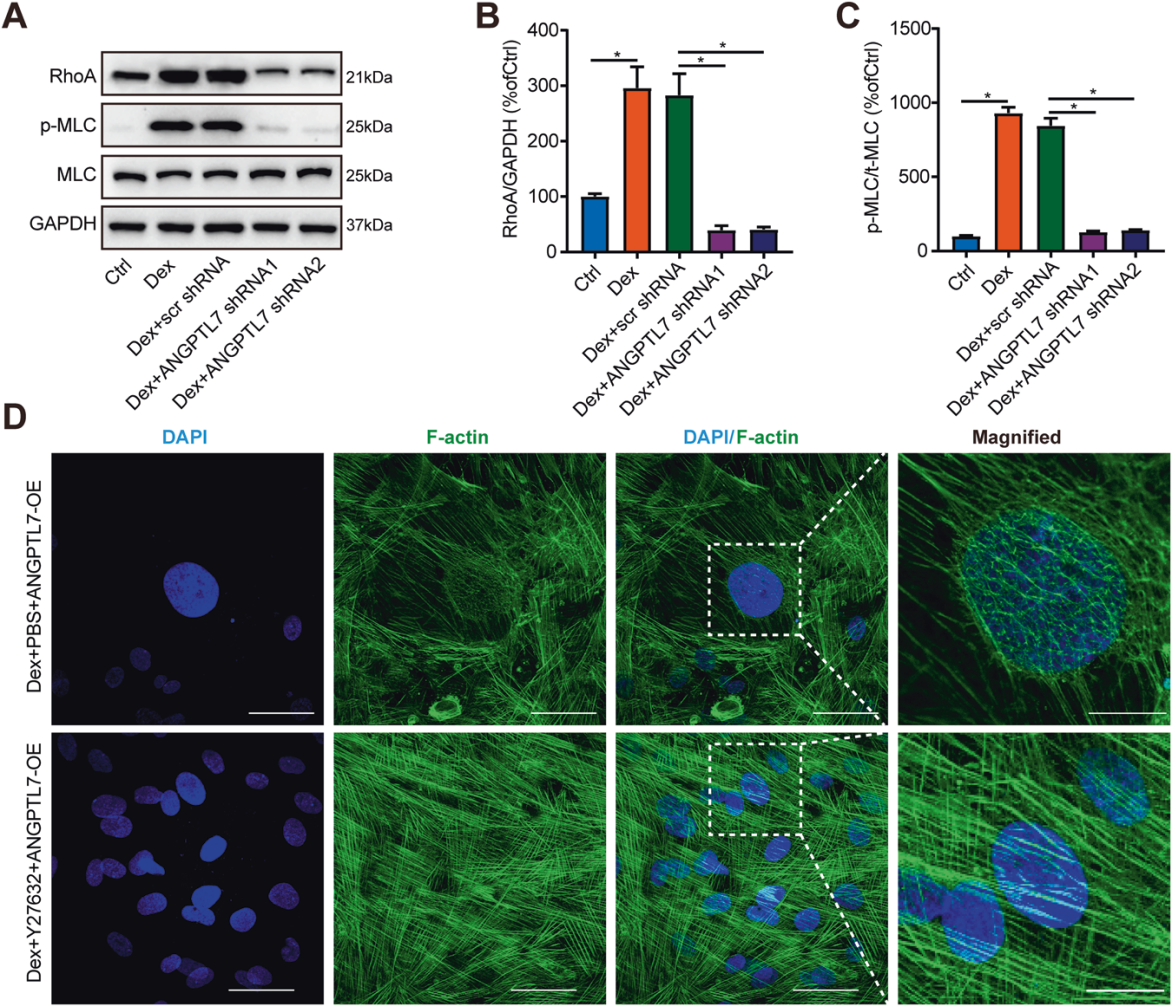
Effects of ANGPTL7 on the RhoA/ROCK pathway. **A**–**C** The expression of critical proteins (RhoA, phosphorylated MLC, and MLC) of the RhoA/ROCK pathway were detected using western blotting in TM cells treated with and without DEX and transfected with scr shRNA and ANGPTL7 shRNAs (shRNA1, shRNA2). The protein levels of RhoA and p-MLC were distinctly increased in TM cells with DEX treatment and dramatically decreased in the DEX + ANGPTL7 shRNAs (shRNA1, shRNA2) group compared with the DEX + scr shRNA group. **D** Representative images of actin filaments of TM cells transfected with ANGPTL7-OE (60× magnification). The insets show regions at higher magnification (200×). ROCK inhibitor Y27632 was used to inhibit the RhoA/ROCK pathway, while PBS was used as a control. Even though ANGPTL7 was overexpressed, the formation of CLANs was suppressed in the DEX + Y27632 + ANGPTL7-OE group compared with the DEX + PBS + ANGPTL7-OE group. Blue: DAPI; green: F-actin. *N* = 3 from three biological replicates. ^*^P < 0.05.

We confirmed the effect of ANGPTL7 on the RhoA/ROCK signal pathway through a series of experiments. First, the protein levels of RhoA and p-MLC were elevated in DEX-treated TM cells. The knockdown of ANGPTL7 evidently reduced the protein expression of RhoA as well as p-MLC (Fig. 4A–C). Moreover, a rescue experiment revealed that ROCK inhibitor Y27632 weakened CLAN formation, even though ANGPTL7 was overexpressed in DEX-treated TM cells (Fig. 4D). These results confirmed that ANGPTL7 modulated formation of CLANs through the RhoA/ROCK pathway.

### SP1 interacted with ANGPTL7 as a transcription factor

**Fig. 5:**
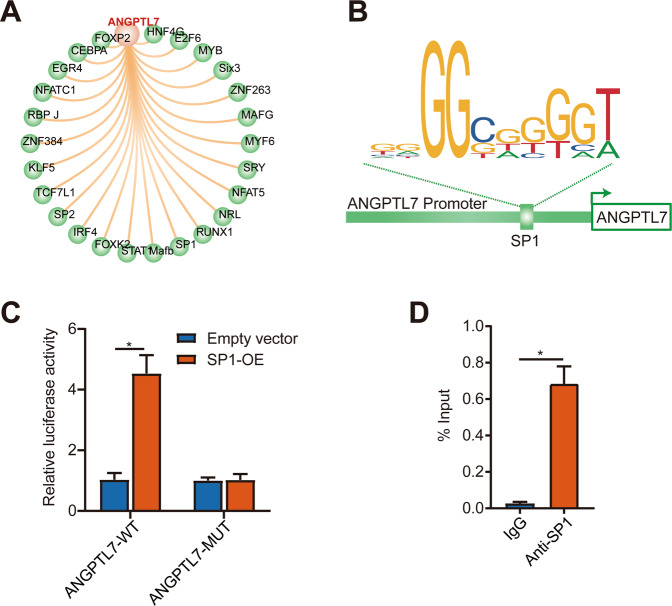
Relationship between SP1 and ANGPTL7. **A** Potential transcription factors of ANGPTL7 predicted using the UCSC Genome Browser. **B** Sites of SP1 predicted to bind to the ANGPTL7 promoter. **C**, **D** The binding relationship between ANGPTL7 and SP1 was examined by dual-luciferase reporter and ChIP assays. *N* = 3 from three biological replicates. ^*^P < 0.05.

To further explore the upstream mechanism of ANGPTL7 in CLAN formation, potential transcription factors of ANGPTL7 were predicted using the UCSC Genome Browser (Fig. 5A) and sites of SP1 to bind to the ANGPTL7 promoter were predicted using the JASPAR database (Fig. 5B). The dual-luciferase reporter assay applied to detect the relationship between ANGPTL7 and SP1 showed that the luciferase activity was markedly upregulated by adopting SP1-OE compared with the empty vector in ANGPTL7-WT reporter vectors. Conversely, the luciferase activity showed no significant changes after co-transfection with an empty vector or SP1-OE in the mutant ANGPTL7 promoter reporter vectors (Fig. 5C). Hence, SP1 was supposed to target the promoter region of ANGPTL7. Furthermore, the ChIP assay indicated that ANGPTL7 DNA fragments were obviously immunoprecipitated by anti-SP1 antibodies in comparison with the control (IgG). In summary, it verified that SP1 protein combined with ANGPTL7 DNA directly (Fig. 5D).

### SP1 mediated ANGPTL7 to regulate DEX-induced CLAN formation

**Fig. 6:**
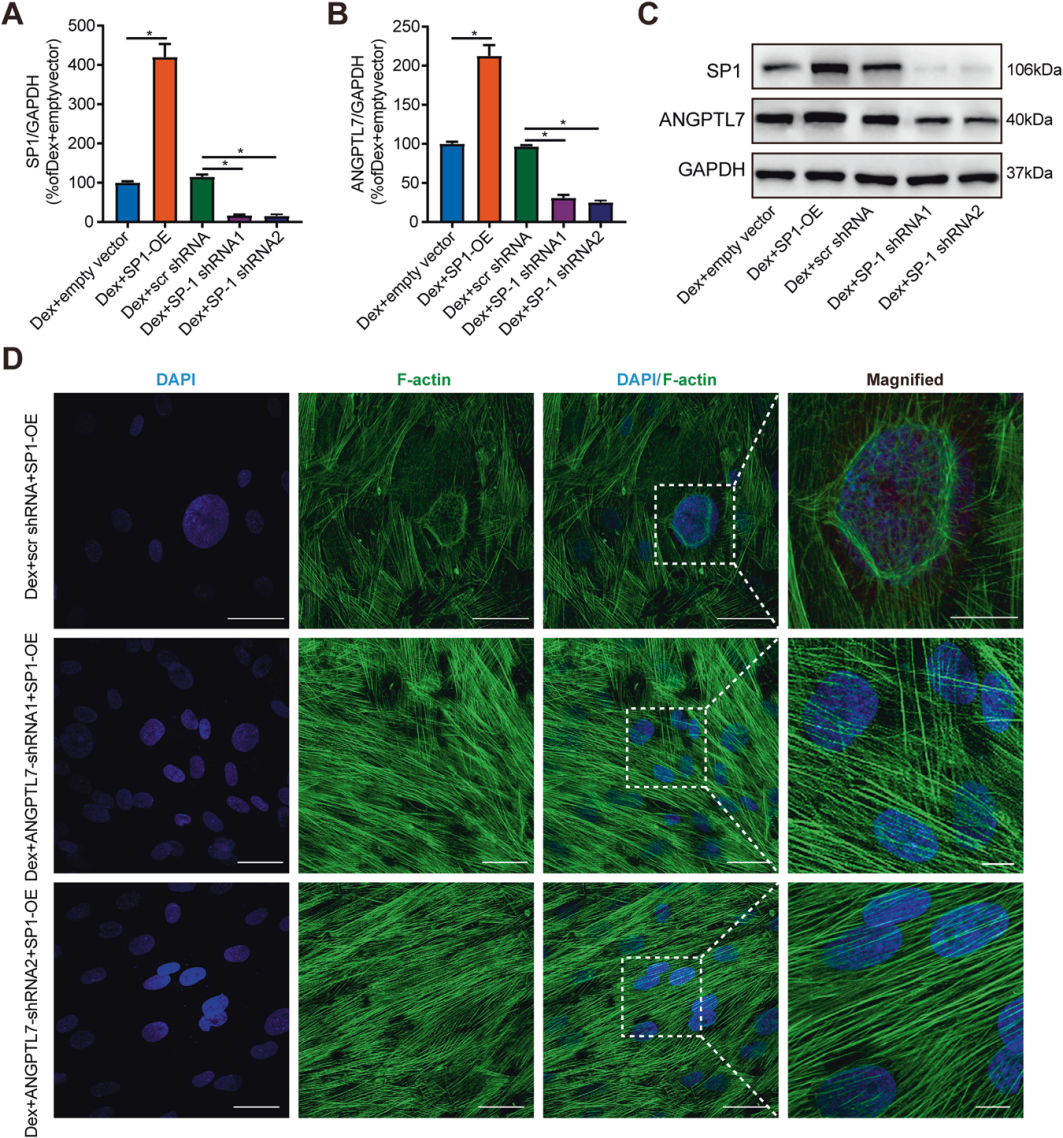
The role of SP1 in the regulation of ANGPTL7. The TM cells were transfected with an empty vector, SP1-OE, SP1 shRNAs (shRNA1, shRNA2), and scr shRNA. **A** The mRNA expression of SP1 and **B** ANGPTL7 assessed by qRT-PCR as well as **C** the protein expression of SP1 and ANGPTL7 assessed by WB showed a marked increase in the DEX + SP1-OE group compared with the DEX + empty vector group and a dramatic decrease in the DEX + SP1 shRNAs (shRNA1, shRNA2) group compared with the DEX + scr shRNA group. **D** Representative images of actin filaments of TM cells transfected with SP1-OE and ANGPTL7 shRNAs (shRNA1, shRNA2) and scr shRNA (60× magnification). The insets show regions at higher magnification (200×). Even though SP1 was overexpressed, the formation of CLANs was distinctly suppressed in the DEX + ANGPTL7 shRNA + SP1-OE group compared with the DEX + scr shRNA + SP1-OE group. Blue: DAPI; green: F-actin. *N* = 3 from three biological replicates. ^*^P < 0.05.

Next, we explored the regulatory effect of SP1 on ANGPTL7. The results showed that SP1 overexpression upregulated the mRNA and protein expressions of SP1 and ANGPTL7 compared with the empty vector group, while SP1 knockdown downregulated the expression of SP1 and ANGPTL7 compared with the scr shRNA group (Fig. 6A–C). This suggested that SP1 induced ANGPTL7 expression. Moreover, a rescue experiment showed that inhibition of ANGPTL7 expression in TM cells with ANGPTL7 shRNAs (shRNA1, shRNA2) effectively suppressed CLAN formation in DEX-induced TM cells, even though SP1 was overexpressed (Fig. 6D). Taken together, the results showed that as a transcription factor, SP1 modulated the expression of ANGPTL7 by directly binding to it and mediated ANGPTL7 to promote DEX-induced CLAN formation.

## Discussion

**Fig. 7:**
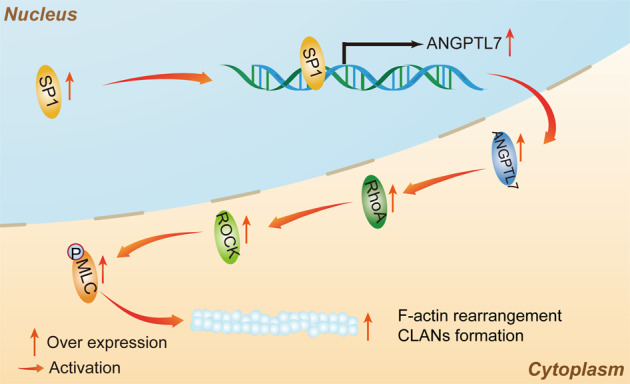
Proposed molecular mechanisms of ANGPTL7 in CLAN formation in TM cells. ANGPTL7 expression is induced by SP1 initiation and activates the RhoA/ROCK signaling pathway to promote CLAN formation in TM cells.

In this study, we detected high levels of ANGPTL7 in DEX-induced TM cells, which promoted the formation of CLANs. Furthermore, ANGPTL7 facilitated CLAN formation by activating the RhoA/ROCK pathway. SP1 was found to mediate the expression of ANGPTL7 as a transcription factor and further modulated CLAN formation (Fig. 7). These discoveries shed novel light on the promoting role of ANGPTL7 in the development of glaucoma and present new targets for its treatment.

Glaucoma remains only partially understood, particularly in the regulation of IOP. Lowering the IOP is currently the only valid approach for controlling the progress of glaucoma [15]. The TM plays an essential role in AH outflow and IOP regulation, in which CLANs are thought to be a major factor contributing to increased outflow resistance [6]. Several studies have found that CLAN formation induced by DEX leads to inhibition of TM cell proliferation, migration, and phagocytosis and causes TM tissue stiffness [8, 16, 17]. However, the specific molecular mechanisms that trigger CLAN formation are rarely known. In this study, we used a previously developed model of DEX-treated TM cells [8] to determine the signaling pathways in CLAN formation. However, the precise lifetime of CLANs could not be confirmed and was assumed to be very long-lived structures [18]. Technical constraints do hinder the tracing of such structures in real time and questions about the lifetime of CLANs remain.

Angiopoietin-like proteins (ANGPTLs) are members of generalized angiopoietin family and are secreted proteins known as angiogenic factors [19]. Some of ANGPTLs were reported to possess the functions of regulating lipid metabolism and inflammation independently of angiogenesis effects [20]. Though ANGPTL7 has been extensively studied, its potential functions in glaucoma have not been characterized. The high inducibility of ANGPTL7 by the glucocorticoid DEX is known [9]. Using bioinformatics analyses and biological experiments, we also found that ANGPTL7 was upregulated by DEX and promoted DEX-induced CLAN formation. Paradoxically, the upregulation of ANGPTL7 is considered a negative regulator of fibronectin expression and has the opposite effect on fibronectin fibril formation associated with POAG in the TM [21]. The reason could be that fibrils in the TM have different conformations and may have different biological properties [22]. Further investigations are needed to examine the association between ANGPTL7 and fibronectin in terms of expression and conformation changes. Besides, overexpression of ANGPTL7 in the TM alters the components of the ECM [9]. Both CLANs formation and ECM remodeling take part in outflow regulation. The effect of ANGPTL7 on AH outflow resistance and IOP regulation needs to be studied.

Based on previous reports [23, 24] and KEGG pathway analysis, we predicted the RhoA/ROCK pathway was the molecular pathway downstream of ANGPTL7. Rho-ROCK signaling involved in cell proliferation, cell differentiation, and apoptosis. Activation of RhoA/ROCK signal pathway has been shown to participate in IOP increase by altering the contractile, cell-adhesive, and stress fiber-formative characteristics of the TM [25, 26]. Moreover, ROCK inhibitors have been shown to significantly increase the outflow facility and lower the IOP by various mechanisms, such as by promoting proliferation and phagocytosis of TM cells, in animal models [27–29]. Importantly, two ROCK inhibitors approved for clinical use (netarsudil in the USA and ripasudil in Japan) have been reportedly effective for the decrease of IOP in glaucoma patients in several clinical trials [30–33]. Consistent with those findings, our data provided evidence that the RhoA/ROCK signaling pathway was involved in TM functions. Besides, we found that ANGPTL7 modulated CLAN formation via the RhoA/ROCK pathway, which acted as a bridge between ANGPTL7 and CLAN formation. We postulated that ROCK inhibition concomitant with ANGPTL7 inhibition may decrease the elevated IOP more effectively. Future work is needed to verify this hypothesis.

To study the exact molecular mechanisms of ANGPTL7 in CLAN formation, we applied the UCSC Genome Browser to predict SP1 as a transcription factor of ANGPTL7 due to its more binding locations and a higher prediction score than those of other transcription factors and a previous report that SP1 activates the molecular pathway of actin cytoskeleton as a transcription factor [12]. We verified that SP1 was a transcription factor of ANGPTL7 by means of dual-luciferase reporter and ChIP assays. Previous studies have reported that the transcription of SP1 apparently mediates biological processes extensively such as cell growth and differentiation, cellular reprogramming, angiogenesis, and tumorigenesis [34]. The role of SP1 in the TM is unknown. In our study, SP1 overexpression and inhibition indicated that SP1 regulated ANGPTL7 expression at both the mRNA and protein levels. A better understanding of the mechanisms underlying SP1 regulation and functions could help determine how ANGPTL7 is transcriptionally regulated by SP1 in CLAN formation, thereby indicating a novel approach targeting SP1 for POAG treatment. The development of either or a combination of ANGPTL7 inhibitors, ROCK inhibitors, and SP1 inhibitors targeted to the CLAN formation and the outflow resistance of TM cells may allow these agents to have great clinical impact.

This is our first attempt to reveal the role of ANGPTL7 and the relevant pathway in CLAN formation. Many questions remain unanswered, and the study is not without limitations. First, the dynamics and functions of CLANs in TM cells are not fully known. It could provide valuable information if live-cell imaging of the actin cytoskeleton were carried out to study the actin dynamics in TM cells. Second, we observed the ultrastructure of CLAN formation by IF staining and qualitatively verified the effect of molecules involved in the signaling pathway. However, a quantitative analysis taking into account the proportion of CLAN-positive cells, the CLAN size, and the dimensions of hubs and spokes would have produced more detailed results. Third, it is uncertain whether IOP elevation is the result or the cause of CLAN formation. Our findings suggest that the former is probably the case. However, the effect of IOP fluctuations on CLAN formation requires further investigation. Moreover, it would be important to confirm the molecular mechanisms using an animal model.

This study shows that ANGPTL7 transcriptionally regulated by SP1 can modulate CLAN formation via the RhoA/ROCK pathway and provides novel insights into how the TM ultrastructure and functions may be altered in some types of glaucoma. Future studies will explore the possibility of regulating the SP1/ANGPTL7/ROCK axis as targeted therapies for steroid-induced glaucoma and POAG.

## Materials and methods

### Identification of differentially expressed genes

The microarray data of GSE65240 were obtained from the publicly available Gene Expression Omnibus (GEO) database (https://www.ncbi.nlm.nih.gov/geo/query/acc.cgi?acc=GSE65240). The datasets consisted of the effects of DEX for primary TM cell gene expression from three lots of TM cells treated with DEX as cases and three lots without DEX as controls. The pheatmap and limma packages of R software were used to draw heat maps and volcano plots, respectively. The R package was applied to calculate the false discovery rate and the log2 fold change (FC). An absolute value of log2 FC > 1 was selected, and an adjusted value of *P* < 0.05 was considered statistically significant.

### KEGG pathway analysis

For Kyoto Encyclopedia of Genes and Genomes (KEGG) pathway enrichment analysis, the clusterProfiler KEGG package of R software was used. A value of *P* < 0.05 was used as the cutoff criterion.

### TM cell culture

Primary TM cells were purchased from ScienCell Research Laboratories (Carlsbad, CA, USA). The cells were cultured in Dulbecco’s modified Eagle’s medium (DMEM; Sigma-Aldrich, Saint Louis, MO, USA) supplemented with 10% fetal bovine serum (Gibco, Invitrogen, Carlsbad, CA, USA), 2 mM L-glutamine (Sigma-Aldrich), 0.05% gentamicin (Mediatech, Herndon, VA, USA), 1% amphotericin B (Mediatech), and 1 ng/mL FGF-2 (PeproTech, Rocky Hill, NJ, USA). The TM cells were grown to confluence. To study the effects of DEX on ANGPTL7 expression, the cells were treated with 100 nM DEX or solvent (ethanol) as a control for 14 days.

### Reagents

The primary antibodies were anti-ANGPTL7 (Abcam, Cambridge, UK), anti-RohA (CST, Beverly, MA, USA), anti-p-MLC (CST), anti-MLC (CST), anti-SP1 (Abcam), phalloidin-488 (Cytoskeleton, Denver, CO, USA), and anti-glyceraldehyde-3-phosphate dehydrogenase (GAPDH) (Abcam). Y27632, a ROCK inhibitor, was purchased from Selleck Chemicals (Houston, TX, USA). Dexamethasone (Sigma D4902) was purchased from Sigma-Aldrich.

### Construction and transfection of short hairpin RNA and overexpression vectors using lentivirus

For ANGPTL7 and SP1 knockdown, short hairpin RNA (shRNA) and scrambled (scr) shRNA were designed and supplied by Genomeditech (Shanghai, China). The lentiviral vectors were transfected into the TM cells according to the manufacturer’s instructions. All the sequences of shRNAs are presented in Table 1. The cells were separately interfered with ANGPTL7/SP1 shRNAs (shRNA1, shRNA2) and scr shRNA and treated for 14 days with DEX. Quantitative reverse transcription–polymerase chain reaction (qRT-PCR) and western blotting (WB) were applied to detect the effects of ANGPTL7/SP1 knockdown.

**Table 1:** The sequence of shRNA.

shRNA	Target sequence (5′-3′)
shRNA negative control	TTCTCCGAACGTGTCACGT
ANGPTL7 shRNA1	GCCATCTACGACTGCTCTTCC
ANGPTL7 shRNA2	GCCCTGAACTGGAGGTGTTCT
SP1 shRNA1	GCAAGTTCTGACAGGACTACC
SP1 shRNA2	GCGTTTCTGCAGCTACCTTGA

For ANGPTL7 and SP1 overexpression, lentiviral overexpression vectors and Lv-empty vectors as controls (PGMLV-CMV-MCS-EF1-mScarlet-T2A-Blasticidin) were also constructed and supplied by Genomeditech. The lentiviral vectors were transfected into the TM cells according to the manufacturer’s instructions. Then 100 nM DEX was added into the medium and the cells were cultured for 14 days.

### Immunofluorescent staining

All cells used to perform immunofluorescent (IF) staining were cultured on coverslips in 12-well plates. Actin filaments (F-actin) were revealed by phalloidin staining. Cells were rinsed with phosphate-buffered saline (PBS) and fixed in 4% paraformaldehyde for 15 min. They were then rinsed with PBS and permeabilized using 0.5% Triton X-100 (Sigma-Aldrich) for half an hour. The cells were rinsed again with PBS and blocked with 1% bovine serum albumin for 1 h. And then the cells were incubated with Alexa Fluor 488-phalloidin (1:500) for 1 h at room temperature. 4′,6-diamidino-2-phenylindole (DAPI) (Sigma-Aldrich) was used for nuclei staining. Imaging was carried out using a confocal microscope (TCS SP8; Leica, Wetzlar, Germany).

### Quantitative reverse transcription PCR

Total RNA was extracted from the TM cells with a Total RNA Extraction Kit (Tiangen, Beijing, China) and reverse transcribed into cDNA with a Synthesis Kit (Takara, Shiga, Japan). QRT-PCR was carried out by using a qPCR Detection Kit (SYBR Green; Tiangen). Gene expression was normalized against GAPDH expression. The 2^−ΔΔCt^ method was used for calculation. All primer pairs are displayed in Table 2.

**Table 2:** Primers used in the qPCR experiments.

Gene	Sequence
ANGPTL7-F	5′-CGGCTGCGTGTAGAGATGGA-3′
ANGPTL7-R	5′-CCTTGGTGCTGAAGGCTGTGT-3′
SP1-F	5′-GAGGGCAGGGTGCCAATG-3′
SP1-R	5′-TTCTGTAAGTTGGGAGCGGC-3′
GAPDH-F	5′-AATCCCATCACCATCTTC-3′
GAPDH-R	5′-AGGCTGTTGTCATACTTC-3′

### Western blotting

Proteins were extracted using a radioimmunoprecipitation assay lysis buffer (ThermoFisher, Pittsburgh, PA, USA) according to standard protocol. Protein concentrations were detected by using a bicinchoninic acid (BCA) protein assay (ThermoFisher). Then, they were isolated using 12% SDS-polyacrylamide gel electrophoresis (SDS–PAGE) and electrotransferred onto polyvinylidene difluoride (PVDF) membranes (Sigma-Aldrich). Blots were blocked by incubation with 5% nonfat milk at room temperature for 1 h and incubated at 4 °C overnight with the following primary antibodies: anti-ANGPTL7 (1:1000), anti-RohA (1:2000), anti-p-MLC (1:2000), anti-MLC (1:2000), anti-SP1 (1:2000), and anti-GAPDH (1:2000). The membranes were rinsed three times and then incubated with the corresponding peroxidase-conjugated secondary antibodies at room temperature for 2 h. An enhanced chemiluminescence system (Millipore, Bedford, MA, USA) was employed to visualize the proteins. Densitometric signal intensity of the western blotting was performed using ImageJ software (http://imagej.nih.gov/ij/; made available in the public domain by the National Institutes of Health, Bethesda, MD, USA).

### Prediction of transcription factors

The underlying transcription factors of ANGPTL7 were identified by employing the UCSC Genome Browser (http://genome.ucsc.edu/). SP1 was chosen since it had more DNA-binding locations and a higher prediction score than other transcription factors. The JASPAR database (http://jaspar.genereg.net/) was used to predict sites where SP1 might bind to the promoter of ANGPTL7.

### Dual-luciferase reporter assay

ANGPTL7 promoter reporter vectors (ANGPTL7-WT) and the corresponding control reporter vectors (ANGPTL7-MUT) were separately obtained from the process that the fragments of ANGPTL7 containing the DNA-binding sites between ANGPTL7 and SP1 or mutation sites were cloned into luciferase reporter vectors (pGL3 vectors; Promega, Madison, WI, USA). These reporter vectors together with SP1 overexpression plasmids (SP1-OE) or corresponding control plasmids (empty vector) were co-transfected into TM cells. The co-transfected cells were lysed and the luciferase activities were assessed using the Dual-Luciferase Reporter Assay System (Promega).

### Chromatin immunoprecipitation (ChIP) assay

The Magnetic ChIP Kit (ThermoFisher) was used to perform chromatin immunoprecipitation according to the manufacturer’s protocol. Anti-SP1 antibodies and control rabbit immunoglobulin G (IgG) were used. After immunoprecipitation, the DNA fragments were purified and the DNA-binding domain of ANGPTL7 with SP1 was detected by using qRT-PCR.

### Statistical analyses

SPSS 17.0 (SPSS, Chicago, IL, USA) was used to perform statistical analyses. The data were expressed as means ± standard deviations from three independently repeated experiments. The paired *t*-test was applied to analyze data between two groups, and one-way ANOVA was applied to analyze results between multiple groups. *P* < 0.05 was considered statistically significant.

## Supplementary information

cddiscovery-author-contribution-form

## Data Availability

All data generated or analyzed during this study are included in this published article. Data sharing is not applicable to this article as no datasets were generated or analyzed during the current study.

## References

[1] Tham YC, Li X, Wong TY, Quigley HA, Aung T, Cheng CY (2014). Global prevalence of glaucoma and projections of glaucoma burden through 2040: a systematic review and meta-analysis. Ophthalmology..

[2] Weinreb RN, Khaw PT (2004). Primary open-angle glaucoma. Lancet..

[3] Clark AF, Wordinger RJ (2009). The role of steroids in outflow resistance. Exp Eye Res..

[4] Clark AF (1995). Basic sciences in clinical glaucoma: steroids, ocular hypertension, and glaucoma. J Glaucoma..

[5] Acott TS, Vranka JA, Keller KE, Raghunathan V, Kelley MJ (2020). Normal and glaucomatous outflow regulation. Prog Retin Eye Res.

[6] Bermudez JY, Montecchi-Palmer M, Mao W, Clark AF (2017). Cross-linked actin networks (CLANs) in glaucoma. Exp Eye Res..

[7] Clark AF, Wilson K, McCartney MD, Miggans ST, Kunkle M, Howe W (1994). Glucocorticoid-induced formation of cross-linked actin networks in cultured human trabecular meshwork cells. Invest Ophthalmol Vis Sci..

[8] Clark AF, Brotchie D, Read AT, Hellberg P, English-Wright S, Pang IH (2005). Dexamethasone alters F-actin architecture and promotes cross-linked actin network formation in human trabecular meshwork tissue. Cell Motil Cytoskeleton..

[9] Comes N, Buie LK, Borrás T (2011). Evidence for a role of angiopoietin-like 7 (ANGPTL7) in extracellular matrix formation of the human trabecular meshwork: implications for glaucoma. Genes Cells..

[10] Kuchtey J, Källberg ME, Gelatt KN, Rinkoski T, Komàromy AM, Kuchtey RW (2008). Angiopoietin-like 7 secretion is induced by glaucoma stimuli and its concentration is elevated in glaucomatous aqueous humor. Invest Ophthalmol Vis Sci..

[11] Tanigawa Y, Wainberg M, Karjalainen J, Kiiskinen T, Venkataraman G, Lemmelä S (2020). Rare protein-altering variants in ANGPTL7 lower intraocular pressure and protect against glaucoma. PLoS Genet..

[12] Yan X, Li M, Luo Z, Zhao Y, Zhang H, Chen L (2020). VIP induces changes in the F-/G-actin ratio of Schlemm’s canal endothelium via LRRK2 transcriptional regulation. Investigative Opthalmology Vis Sci..

[13] Wang J, Liu X, Zhong Y (2013). Rho/Rho-associated kinase pathway in glaucoma. Int J Oncol.

[14] Yuan Y, Call MK, Yuan Y, Zhang Y, Fischesser K, Liu CY (2013). Dexamethasone induces cross-linked actin networks in trabecular meshwork cells through noncanonical wnt signaling. Invest Ophthalmol Vis Sci..

[15] Sultan MB, Mansberger SL, Lee PP (2009). Understanding the importance of IOP variables in glaucoma: a systematic review. Surv Ophthalmol..

[16] Zhang X, Ognibene CM, Clark AF, Yorio T (2007). Dexamethasone inhibition of trabecular meshwork cell phagocytosis and its modulation by glucocorticoid receptor beta. Exp Eye Res..

[17] Fujimoto T, Inoue T, Inoue-Mochita M, Tanihara H (2016). Live cell imaging of actin dynamics in dexamethasone-treated porcine trabecular meshwork cells. Exp Eye Res..

[18] Duffy L, O’Reilly S (2018). Functional implications of cross-linked actin networks in trabecular meshwork cells. Cell Physiol Biochem..

[19] Niki D, Katsu K, Yokouchi Y (2009). Ontogeny of angiopoietin-like protein 1, 2, 3, 4, 5, and 7 genes during chick embryonic development. Dev Growth Differ..

[20] Qian T, Wang K, Cui J, He Y, Yang Z (2016). Angiopoietin-like protein 7 promotes an inflammatory phenotype in RAW264.7 macrophages through the P38 MAPK signaling pathway. Inflammation..

[21] Faralli JA, Filla MS, Peters DM (2019). Role of fibronectin in primary open angle glaucoma. Cells..

[22] Antia M, Baneyx G, Kubow KE, Vogel V (2008). Fibronectin in aging extracellular matrix fibrils is progressively unfolded by cells and elicits an enhanced rigidity response. Faraday Discuss..

[23] Pattabiraman PP, Inoue T, Rao PV (2015). Elevated intraocular pressure induces Rho GTPase mediated contractile signaling in the trabecular meshwork. Exp Eye Res..

[24] Liesenborghs I, Eijssen LMT, Kutmon M, Gorgels T, Evelo CT, Beckers HJM (2020). Comprehensive bioinformatics analysis of trabecular meshwork gene expression data to unravel the molecular pathogenesis of primary open-angle glaucoma. Acta Ophthalmol..

[25] Riento K, Ridley AJ (2003). Rocks: multifunctional kinases in cell behaviour. Nat Rev Mol Cell Biol..

[26] Rao PV, Pattabiraman PP, Kopczynski C (2017). Role of the Rho GTPase/Rho kinase signaling pathway in pathogenesis and treatment of glaucoma: Bench to bedside research. Exp Eye Res..

[27] Wang J, Liu X, Zhong Y (2013). Rho/Rho-associated kinase pathway in glaucoma (review). Int J Oncol..

[28] Inoue T, Tanihara H (2013). Rho-associated kinase inhibitors: a novel glaucoma therapy. Prog Retin Eye Res..

[29] Chen W, Yang X, Fang J, Zhang Y, Zhu W, Yang X (2020). Rho-associated protein kinase inhibitor treatment promotes proliferation and phagocytosis in trabecular meshwork cells. Front Pharmacol..

[30] Maruyama Y, Ikeda Y, Mori K, Yoshii K, Ueno M, Sotozono C (2020). Safety and efficacy of long-term ripasudil 0.4% instillation for the reduction of intraocular pressure in Japanese open-angle glaucoma patients. J Ocul Pharm Ther..

[31] Kusuhara S, Nakamura M (2020). Ripasudil hydrochloride hydrate in the treatment of glaucoma: safety, efficacy, and patient selection. Clin Ophthalmol..

[32] Sit AJ, Gupta D, Kazemi A, Mckee H, Challa P, Liu KC (2021). Netarsudil improves trabecular outflow facility in patients with primary open angle glaucoma or ocular hypertension: a phase 2 study. Am J Ophthalmol.

[33] Tanna AP, Johnson M (2018). Rho kinase inhibitors as a novel treatment for glaucoma and ocular hypertension. Ophthalmology..

[34] Vellingiri B, Iyer M, Devi Subramaniam M, Jayaramayya K, Siama Z, Giridharan B (2020). Understanding the role of the transcription factor Sp1 in ovarian cancer: from theory to practice. Int J Mol Sci..

