# Supplementary material for: ANGPTL7 is transcriptionally regulated by SP1 and modulates glucocorticoid-induced cross-linked actin networks in trabecular meshwork cells via the RhoA/ROCK pathway
Source: Cell Death Discov. 2022 Feb 8;8:50. doi: 10.1038/s41420-022-00847-3 (PMC8826420; doi:10.1038/s41420-022-00847-3)
Supplement: Supplementary file 1 — cddiscovery-author-contribution-form [file 41420_2022_847_MOESM1_ESM.pdf]

DECLARATION OF CONTRIBUTIONS TO ARTICLE

ADMC

Manuscript Number:	Journal Name:	
CDDISCOVERY-21-1710	*Cell Death Discovery*	(the 'Journal')

Proposed Title of the Contribution:

ANGPTL7 is transcriptionally regulated by SP1 and modulates Glucocorticoid-induced Cross-Linked Actin Networks in Trabecular Meshwork Cells via the RhoA/ROCK pathway (the 'Contribution)

Author(s):

Mengsha Sun, Wenjia Liu, Minwen Zhou (the 'Authors')

For all *CDDis*covery articles, each person named as an author in the published version must be able to show he or she has contributed substantially to the article.

Authorship credit should be based on 1) substantial contributions to conception and design, acquisition of data, or analysis and interpretation of data; 2) drafting the article or revising it critically for important intellectual content; and 3) final approval of the version to be published. Authors should meet conditions 1, 2 and 3.

Any person who cannot be shown to have made a substantial contribution to the article cannot be listed as an author in the final version. The name of any person who is deemed to have made a minor contribution can, however, appear in the Acknowledgments section of the article.

Please complete the table below to indicate the contributions of all named authors to the manuscript.

Author Full Name:	Specification of Contribution to the Manuscript:
Mengsha Sun	contributions to conception and design, acquisition of data, analysis and interpretation of data; drafting the article; final approval of the version to be published.
Wenjia Liu	contributions to conception and design, analysis and interpretation of data; revising it critically for important intellectual content; final approval of the version to be published.
Minwen Zhou	contributions to conception and design, acquisition of data, analysis and interpretation of data; revising it critically for important intellectual content; final approval of the version to be published.

Please complete the table below to indicate the contributions of all named authors to the figures.

Figure 1:

MS and MZ contributed to the conception and design of the study. MS and WL acquired the data and performed the statistical analysis. MZ interpreted the data.

Figure 2:

MS and MZ contributed to the conception and design of the study. MS and WL acquired the data and performed the statistical analysis. MS and MZ interpreted the data.

Figure 3:

MS, WL and MZ contributed to the conception and design of the study. MS and WL performed the statistical analysis. MS and MZ interpreted the data.

Figure 4:

MS and MZ contributed to the conception and design of the study. MS and WL acquired the data and performed the statistical analysis. MZ interpreted the data.

Figure 5:

MS and MZ contributed to the conception and design of the study. MS and WL performed the statistical analysis. MZ interpreted the data.

Figure 6:

MS and MZ contributed to the conception and design of the study. MS and WL acquired the data and performed the statistical analysis. MS and MZ interpreted the data.

Signed for and on behalf of the Author(s):	Print Name:	Date:
	Minwen Zhou	2021-04-29
